# Hyperprogressive Disease After Combined Anti-PD-L1 and Anti-CTLA-4 Immunotherapy for MSI-H/dMMR Gastric Cancer: A Case Report

**DOI:** 10.3389/fonc.2021.756365

**Published:** 2021-09-24

**Authors:** Romain Varnier, Thibaut Garrivier, Emilie Hafliger, Aymeric Favre, Clélia Coutzac, Clément Spire, Pauline Rochefort, Matthieu Sarabi, Françoise Desseigne, Pierre Guibert, Anne Cattey-Javouhey, Pamela Funk-Debleds, Charles Mastier, Adrien Buisson, David Pérol, Oliver Trédan, Jean-Yves Blay, Jean-Marc Phelip, Christelle de la Fouchardiere

**Affiliations:** ^1^ Department of Medical Oncology, Centre Léon Bérard, Lyon, France; ^2^ Department of Radiology, Centre Léon Bérard, Lyon, France; ^3^ Department of Biopathology, Centre Léon Bérard, Lyon, France; ^4^ Department of Clinical Research, Centre Léon Bérard, Lyon, France; ^5^ Department of Hepato-Gastroenterology and Digestive Oncology, St Etienne University Hospital, St Etienne, France

**Keywords:** gastric cancer, MSI -H, immunotherapy, hyperprogression, durvalumab, tremelimumab, case report

## Abstract

Immune checkpoint inhibitors (ICI) have been developed in gastric adenocarcinomas and approved in first-line metastatic setting (in combination with chemotherapy) as well as in pretreated patients. Microsatellite instability-high (MSI-H) tumors are predicted to derive high benefit from ICI but data in gastric locations are limited. Here, we describe the case of a 68-year old patient with stage IV MSI-H gastric adenocarcinoma, referred to our center to receive immunotherapy after failure of standard of care (surgery with perioperative platin-based chemotherapy and paclitaxel plus ramucirumab at disease progression). The patient received one injection of durvalumab and tremelimumab and was hospitalized eighteen days after because of occlusive syndrome. The CT scan showed hyperprogression of the lymph nodes and hepatic lesions, compressing the gastric stump. He died few days later. Molecular analyses did not explain this outcome. To our knowledge, this is one of the first reported cases of hyperprogressive disease after combined ICI for a patient with MSI-H tumor. We review the potential causes and discuss the emerging literature regarding predictive factors of hyperprogression in the particular subset of MSI-H patients. If some data were available in retrospective studies, validation of strong predictive factors is needed to avoid such dramatic evolutions.

## Introduction

Despite progresses in prevention and screening, gastric adenocarcinoma (GA) remains the third cause of cancer-related mortality worldwide (1). Immune checkpoint inhibitors (ICI) have been developed and evaluated in several settings in GA. The benefit of nivolumab and pembrolizumab, two PD-1 inhibitors, was first shown over placebo in pretreated patients in the ATTRACTION-2 and KEYNOTE-061 trials (2, 3). In the first-line metastatic setting, addition of nivolumab to chemotherapy recently improved overall survival (14.4 *versus* 11.1 months) for PD-L1-positive [combined positive score (CPS) ≥ 5] advanced gastric and esophageal adenocarcinomas (4).

Somatic genomic analysis of large series of gastric cancers identified distinct molecular subtypes with their own prognosis and therapeutic targets. The Cancer Genome Atlas classified 22% of gastric cancers as microsatellite instability-high (MSI-H) tumors, with deficient mismatch repair (dMMR) and high mutational burden (TMB) (5). As for colorectal adenocarcinoma, MSI-H tumors seem to have a good prognosis, and are more frequent in localized gastric cancers (6 to 20%) (6–10) than in advanced disease (2.5-3%) (10, 11).

The benefit of ICI for MSI-H tumors was first provided by a small phase 2 trial evaluating pembrolizumab in refractory tumors with or without MMR deficiency (12). Efficacy was shown in both colorectal and non-colorectal MSI-H/dMMR tumors including one MSI-H gastric cancer. The KEYNOTE-158 study further confirmed the benefit of pembrolizumab for various non-colorectal MSI-H/dMMR tumors, with a 46% response rate and a 11 months median progression-free survival for the gastric adenocarcinoma subgroup (13). Later, an exploratory analysis of the KEYNOTE-062 study showed that pembrolizumab improved overall survival compared to first-line chemotherapy in the small MSI-H subgroup (50/763 patients) whereas it was only non-inferior for the overall population of PD-L1-positive (CPS ≥ 1) gastric adenocarcinomas (14, 15). A recently published meta-analysis of randomized clinical trials confirmed the predictive role of microsatellite instability for PD-1 blockade efficacy (16). However, the number of MSI-H GA treated with ICI is currently low (all available data in MSI-H GA are summarized in **Table 1**).

**Table 1:** Results of MSI-H GA patients treated with ICI monotherapy.

Study	Line	Number of MSI GA	ORR (95% CI)	PFS (95% CI)	OS (95% CI)
**KEYNOTE-158**	Pembrolizumab ≥ L2	24	45.8% (25.6-67.2)	11.0 months (2.1-NR)	NR (7.2 months-NR)
Marabelle et al. (13)					
**KEYNOTE-062**	Pembrolizumab L1	14/256	57.1% (NA)	11.2 months (NA)	NR (10.7 months-NR)
Shitara et al. (15)					
**KEYNOTE-061**	Pembrolizumab L2	15/296	46.7% (NA)	17.8 months (NA)	NR (5.6 months-NR)
Shitara et al. (3)					

ORR, overall response rate; PFS, progression-free survival; OS, overall survival; NR, not reached; NA, not available.

Patterns of response and progression during immunotherapy may differ from what is observed with chemotherapy (17). A dramatic worsening of disease progression, known as hyperprogressive disease (HPD) has been described in a subset of patients treated with immunotherapy, especially for head and neck squamous cell (18) and non-small cell lung cancers (19), but data are lacking about HPD in MSI-H tumors for which only a few cases have been reported (20, 21).

We described the case of a MSI-H/dMMR gastric adenocarcinoma patient with HPD while receiving ICI, and reviewed the potential causes and predictive factors of hyperprogression.

## Case Presentation

We report the case of a 68-year old Caucasian patient with a metastatic MSI-H/dMMR gastric adenocarcinoma. This patient with no significant past medical history was treated four years ago for localized antral gastric cancer. He benefited from a subtotal gastrectomy with perioperative FOLFOX chemotherapy (5 cycles received). Pathological examination of surgical specimen revealed poorly differentiated GA, classified ypT3N1 (2N+/23), HER2-negative [immunohistochemistry (IHC) 2+, fluorescence in-situ hybridization (FISH) negative], Epstein-Barr Virus ambiguous status (positive on the first sample but not confirmed on the second sample), *Helicobacter pylori*-negative, with MMR-deficiency and microsatellite-instability (loss of expression of MLH1 and PMS2, caused by MLH1 promoter hypermethylation).

The patient experienced early recurrence in retroperitoneal and intraperitoneal lymph nodes after ending the perioperative chemotherapy. He received paclitaxel and ramucirumab as second-line chemotherapy, with a near-complete tumor response, followed by radiochemotherapy on residual disease.

After two years of follow-up, he presented another locoregional lymph node recurrence and was treated again with paclitaxel and ramucirumab until progression (apparition of hepatic lesions and increase of lymph nodes) occurring 7 months later.

Due to the dMMR/MSI-H status and lack of immunotherapy approval in France for non-colorectal MSI-H cancers, he was referred to our comprehensive cancer center. His general condition was good (ECOG-PS 1) but he presented a 11% weight loss during the previous 6 months. Blood analysis showed mild perturbations of hepatic function, twofold elevation of LDH and elevated neutrophil-to-lymphocyte ratio (5.7) (**Table 2**). After discussion in multidisciplinary tumor board and written consent, the patient was enrolled in the immunotherapy cohort of the “MOST plus” phase II trial (NCT02029001) evaluating the benefit of the PD-L1 inhibitor durvalumab (1500 mg flat dose every 4 weeks until disease progression) combined with the CTLA4 inhibitor tremelimumab (1 mg/kg every 4 weeks for 4 cycles) in immunogenic tumors such as MSI-H or high mutational board tumors after failure of standard of care.

**Table 2:** Chronological evolution of clinical and biological parameters.

Relevant Past Medical History and Interventions		
**68 years old Caucasian patient** without significant comorbidity.		
Treated four years ago by **subtotal gastrectomy with perioperative FOLFOX chemotherapy** for a **localized gastric cancer** (ypT3N1). Histology: poorly differentiated adenocarcinoma, HER2-negative, EBV-ambiguous, **dMMR/MSI-H, PD-L1 < 1%**). RNAseq analysis: deleterious **TP53 mutation**.		
Retroperitoneal **lymph node recurrence** treated with **paclitaxel ramucirumab** followed by **radiochemotherapy**.		
Second **lymph node recurrence** treated again with **paclitaxel ramucirumab until progression** (apparition of hepatic lesions).		
Enrollment in the **“MOST plus” trial** to receive **durvalumab tremelimumab** combination.		
	**Visit dates**	
	**July 6^th^ (first cycle)**	**July 24^th^ (hospitalization)**
Physical examination		
Weight (kg)	76	75
ECOG	PS 1	PS 3
Laboratory biomarkers		
*Liver function*		
ASAT (U/L)	63	242
ALAT (U/L)	45	109
gammaGT (U/L)	283	1772
PAL (U/L)	381	2840
Total bilirubin (µmol/L)	4	17
*Inflammation and tumor burden markers*		
CRP (mg/L)	–	150
White blood cell count (G/L)	6.4	8.2
Absolute neutrophil count (G/L)	4.7	6.8
Absolute lymphocyte count (G/L)	0.82	0.67
NLR ratio	5.7	10.1
dNLR ratio	2.76	4.86
LDH (U/L)	638	1072
LIPI score	2	2
CAE (ng/mL)	< 5	20.9
**Death on August 5^th^**		

**Figure 1:**
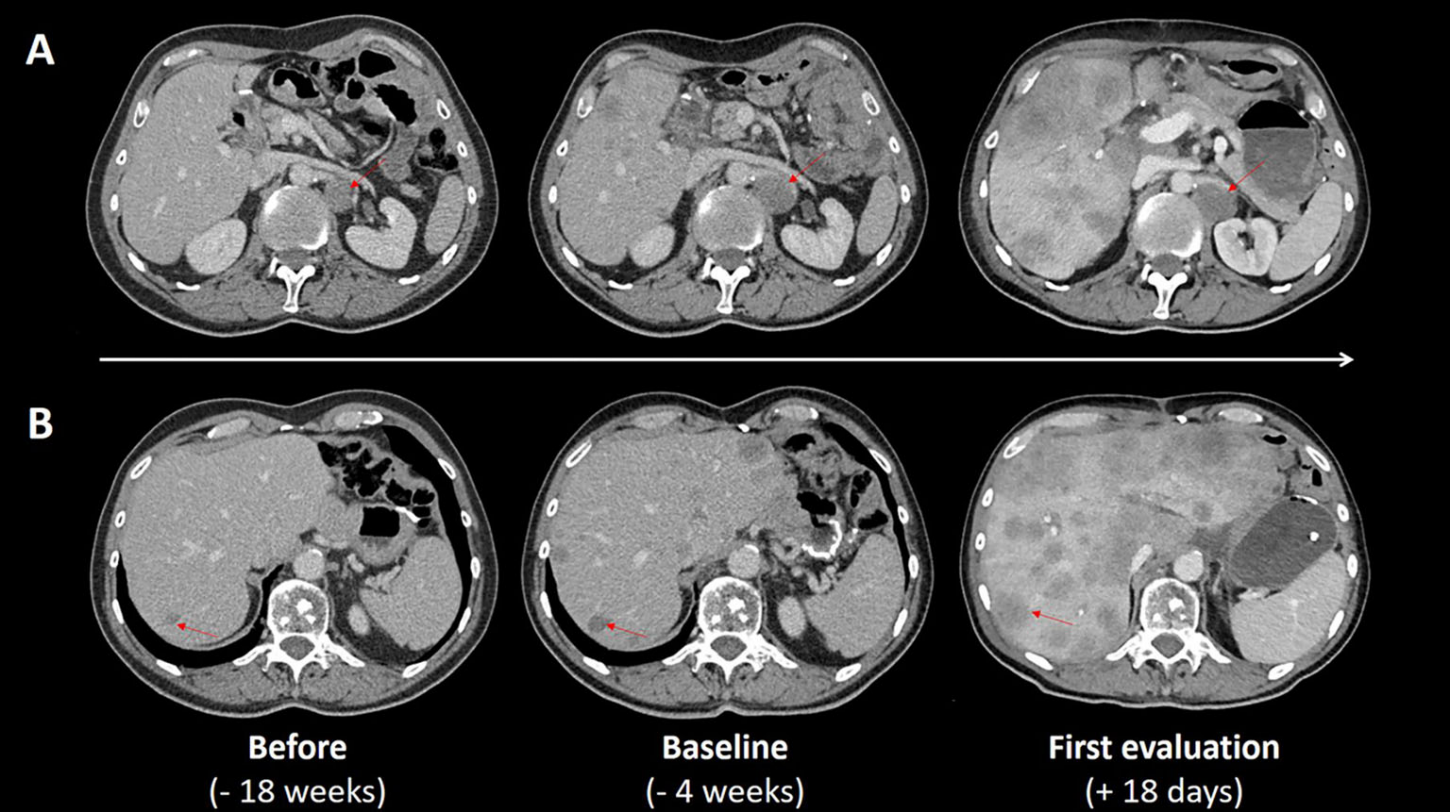
CT scan evaluations before and after treatment. CT scans performed 18 weeks before ICI treatment (first column), at baseline (second column) and for early evaluation eighteen days after the beginning of ICI treatment (third column) show the changes in lymph nodes and hepatic lesions: the left para-aortic lymph node (red arrow, line **A**) increased from 30mm at baseline to 40mm on day 18, known hepatic lesion increased (red arrow, line **B**), multiple new hepatic lesions appeared and locoregional invasion led to gastric stump compression.

The patient was hospitalized in emergency eighteen days after the first injection because of intestinal obstruction and impaired general condition. The CT-scan showed significant disease progression of lymph nodes and hepatic lesions, compressing the gastric stump (**Figure 1**). Laboratories exams showed grade 3 cytolysis, anicteric cholestasis, rising of neutrophil-to-lymphocyte ratio (to 10.1) and LDH and elevation of tumor marker ACE to 20.9 ng/mL (normal before treatment) (**Table 2**). The situation worsened rapidly with liver impairment evolving to hepatic encephalopathy and death twelve days later.

Hyperprogression was confirmed by an external review of an expert radiologist calculating the Tumor Growth Kinetics ratio (TGKR) to 7 (**Figure 1**).

After this unexpected outcome, we decided to repeat molecular analyses on a liver metastasis biopsy performed a few days before starting immunotherapy. The expert pathologist confirmed the lack of expression of MLH1 and PMS2 proteins with IHC [antibodies anti-MSH2 (clone FE11, Dako), anti-MSH6 (clone EP49, Dako), anti-MLH1 (clone ES05, Dako) and anti-PMS2 (clone EP51, Dako) on Benchmark Ultra; detection kit Ultraview Universal DAB ref: 760-500, Amplification kit Ref 760-080, with positive internal controls] and evaluated the PD-L1 expression in tumor cells as negative (< 1% of membrane expression; clone SP263, Kit Ventana Ref 790-4905 on Benchmark Ultra; detection kit Ultraview Universal DAB ref: 760-500, Amplification kit Ref 760-080, with positive external controls). RNAseq analysis identified a deleterious mutation in TP53 gene (FusionPlex RNA CTL_V6, Archerdx; list of covered genes in **Supplementary Table 1**). No other significant molecular alteration was identified and FISH confirmed the lack of MDM2 amplification (ZytoVision ZytoLight SPEC MDM2/CEN 12 Dual Color Probe).

## Discussion

### Hyperprogression Definition

Evaluation of therapeutic response to immunotherapy can be more challenging than with conventional cytotoxic therapy, given the different patterns of tumor response. Pseudoprogressions (PSPD) – with initial flare-up followed by prolonged responses – must be differentiated from hyperprogressions in which the tumor increase continues (22). Several radiologic criteria were developed to define HPD (19). Le Tourneau et al. defined Tumor Growth Kinetics (TGK) as the change in the tumor size per unit of time (in mm/d) (23). Saâda-Bouzid et al. then defined a TGK ratio (TGKR) comparing the post-immunotherapy TGK to the pre-immunotherapy TGK: a TGKR> 1 indicated tumor growth acceleration and a TGKR ≥ 2 defined hyperprogression (18). Recently, Colle et al. reviewed their cases of MSI/dMMR metastatic colorectal cancers treated with ICIs and identified PSPD in 10% of their population, occurring early (within the first 3 months) and accompanied with a clinical benefit and a biological response (CEA) (24). In the present clinical case, RECIST 1.1 progression associated with clinical impaired condition and biological hepatic perturbations were in favor of HPD and not PSPD.

### Hyperprogression in Gastric Cancers: Epidemiology and Predictive Factors

Several clinical factors were found to be associated with the likelihood of HPD among various tumors. These include advanced age, cancer recurrence, prior radiation therapy, increased metastatic burden and liver metastasis (25, 26).

Hyperprogressive disease for MSI-H/dMMR gastric cancer has never been reported. A few cases have been reported for gastric cancers (27–31) but not specifically for MSI-H tumors. Retrospective cohort studies reported hyperprogressive diseases rates of 10% to 29% in common gastric cancers treated with nivolumab (32–35). Impaired general condition and liver metastasis were associated with HPD in two of these studies (33, 35). A large sum of target lesion diameters at baseline (35) and a PD-L1 CPS score < 10 (33) were reported as potential predictive factors of hyperprogression. These studies also confirmed the better prognosis and higher response rate to immunotherapy for MSI-H gastric cancers. HPD rates were about 12-14% in MSI-H tumors and 23% in proficient-MMR tumors, but there was no statistical association in these small samples (only four MSI-H hyperprogressors) (33, 35).

While having a confirmed MSI-H GA, the tumor in our case was PD-L1-negative, maybe explaining the lack of ICI efficacy. However, MSI-H tumors are associated with high numbers of infiltrating lymphocytes which can mediate antitumor response, even in PD-L1-negative tumors (36, 37). Another predictive biomarker for ICI efficacy is tumor mutational burden (TMB): pembrolizumab recently obtained an FDA approval for TMB-H cancer treatment following the results of the KEYNOTE-158 study (38). However, no gastric cancer was included in this study and the TMB evaluation is not yet standardized. An interesting report showed substantial overlap between MSI and TMB-high tumors in 63 gastric cancer patients treated with ICI with only one non-responder MSI-H GA patient, bearing a PD-L1-positive but low-TMB tumor (39).

Several scores were developed to predict ICI efficacy. The Lung Immune Prognostic Index (LIPI) is a simple tool which stratifies patients in “poor”, “intermediate” and “good” prognostic groups according to pre-treatment LDH (one point if greater than the upper limit of normal, defined according the limit of local laboratory) and derived neutrophil-to-lymphocyte ratio (dNLR = absolute neutrophil count/[white blood cell concentration − absolute neutrophil count]; one point if ≥ 3) (40). This score was initially developed for advanced non-small cell lung cancers and further validated in renal cell carcinoma, melanoma and gastric cancer (41, 42). A multicenter retrospective analysis of patients with metastatic MSI-H/dMMR tumors treated with ICI showed that “poor” LIPI score was significantly associated with shorter survival and higher rate of fast-progression (defined by ≤ 3 months overall survival) (43). In our case, the pre-treatment LIPI score was “intermediate”. The LIPI score could be useful to identify patients at high-risk of fast-progression but prospective validation with recognized hyperprogression criteria is needed.

### Causes of Hyperprogression

Pathological mechanisms for hyperprogressive disease are being actively investigated. Analysis of hyperprogressive gastric cancers showed that ICI could increase Treg cell infiltration and therefore enhance their immunosuppressive abilities (34). Infiltration and activation of M2 macrophages has also been associated with hyperprogressive disease (44). However, these results were only partially reproducible in Yamaguchi et al. case report of a patient with PD-L1 negative metastatic gastric cancer who presented hyperprogressive disease after nivolumab third line therapy: tissue sample analysis of the hyperprogressive lymph node showed PD-L1-positive macrophage increase, but also Treg decrease which was unexpected (45). In our case, we were not able to perform a liver biopsy to evaluate immune infiltration after failure of ICI because the patient’s general condition worsened too quickly.

Genomic analysis of hyperprogressive tumors identified an overrepresentation of EGFR alterations and MDM2/MDM4 amplifications among them (46). In our case, none of these molecular alterations was identified.

## Conclusion

Microsatellite instability is a strong predictive factor for tumor response, regardless of tumor site of origin. However, this case reminds us that MSI-H status does not guarantee a response to ICI: even in this highly-selected population, disease progression and even hyperprogression can be observed. Despite recent efforts, the prevalence, mechanisms, and predictive factors for HPD remain unclear.

We report here the first case of HPD in a PD-L1-negative MSI-H gastric cancer patient treated with an ICI combination and describe in detail its clinical, biological and molecular characteristics. Combining MMR status with TMB and PD-L1 analyses may be of great interest to identify ICI-responders. Validation of a combined score or other predictive factors is needed to avoid such dramatic evolutions with ICI.

## Data Availability Statement

The datasets presented in this study can be found in online repositories. The names of the repository/repositories and accession number(s) can be found below: https://www.ncbi.nlm.nih.gov/bioproject/PRJNA755252.

## Ethics Statement

Written informed consent was obtained from the individual(s) for the publication of any potentially identifiable images or data included in this article.

## Author Contributions

RV and AF collected data. TG and CM reviewed CT scans and calculated the TGK ratio. AB performed the molecular analyzes. RV and CF wrote the initial manuscript. All authors contributed to the article and approved the submitted version.

## Funding

LYRICAN (INCA-DGOS-INSERM 12563), LabEx DEvweCAN (ANR-10-LABX-0061), Institut Convergence PLASCAN (17-CONV-0002), RHU4 DEPGYN (ANR-18-RHUS-0009), Association DAM’s, NetSARC+ (INCA & DGOS), EURACAN (EC 739521), la Fondation ARC (PGA 1° 2016 02 03 721), La Ligue contre le Cancer, funded this study.

## Conflict of Interest

The authors declare that the research was conducted in the absence of any commercial or financial relationships that could be construed as a potential conflict of interest.

## Publisher’s Note

All claims expressed in this article are solely those of the authors and do not necessarily represent those of their affiliated organizations, or those of the publisher, the editors and the reviewers. Any product that may be evaluated in this article, or claim that may be made by its manufacturer, is not guaranteed or endorsed by the publisher.

## References

[1] Bray F Ferlay J Soerjomataram I Siegel RL Torre LA Jemal A. Global Cancer Statistics 2018: GLOBOCAN Estimates of Incidence and Mortality Worldwide for 36 Cancers in 185 Countries. CA: A Cancer J Clin (2018) 68:394–424. doi: 10.3322/caac.21492 30207593

[2] Kang Y-K Boku N Satoh T Ryu M-H Chao Y Kato K. Nivolumab in Patients With Advanced Gastric or Gastro-Oesophageal Junction Cancer Refractory to, or Intolerant of, at Least Two Previous Chemotherapy Regimens (ONO-4538-12, ATTRACTION-2): A Randomised, Double-Blind, Placebo-Controlled, Phase 3 Trial. Lancet (2017) 390:2461–71. doi: 10.1016/S0140-6736(17)31827-5 28993052

[3] Shitara K Özgüroğlu M Bang Y-J Di Bartolomeo M Mandalà M Ryu M-H. Pembrolizumab *Versus* Paclitaxel for Previously Treated, Advanced Gastric or Gastro-Oesophageal Junction Cancer (KEYNOTE-061): A Randomised, Open-Label, Controlled, Phase 3 Trial. Lancet (2018) 392:123–33. doi: 10.1016/S0140-6736(18)31257-1 29880231

[4] Janjigian YY Shitara K Moehler M Garrido M Salman P Shen L. First-Line Nivolumab Plus Chemotherapy *Versus* Chemotherapy Alone for Advanced Gastric, Gastro-Oesophageal Junction, and Oesophageal Adenocarcinoma (CheckMate 649): A Randomised, Open-Label, Phase 3 Trial. Lancet (2021) 398:27–40. doi: 10.1016/S0140-6736(21)00797-2 34102137 PMC8436782

[5] Bass AJ Thorsson V Shmulevich I Reynolds SM Miller M Bernard B. Comprehensive Molecular Characterization of Gastric Adenocarcinoma. Nature (2014) 513:202–9. doi: 10.1038/nature13480 PMC4170219 25079317

[6] An JY Kim H Cheong J-H Hyung WJ Kim H Noh SH. Microsatellite Instability in Sporadic Gastric Cancer: Its Prognostic Role and Guidance for 5-FU Based Chemotherapy After R0 Resection. Int J Cancer (2012) 131:505–11. doi: 10.1002/ijc.26399 21898388

[7] Beghelli S de Manzoni G Barbi S Tomezzoli A Roviello F Di Gregorio C. Microsatellite Instability in Gastric Cancer Is Associated With Better Prognosis in Only Stage II Cancers. Surgery (2006) 139:347–56. doi: 10.1016/j.surg.2005.08.021 16546499

[8] Bonneville R Krook MA Kautto EA Miya J Wing MR Chen H-Z. Landscape of Microsatellite Instability Across 39 Cancer Types. JCO Precis Oncol (2017) 1. doi: 10.1200/PO.17.00073 PMC5972025 29850653

[9] Kim H An JY Noh SH Shin SK Lee YC Kim H. High Microsatellite Instability Predicts Good Prognosis in Intestinal-Type Gastric Cancers. J Gastroenterol Hepatol (2011) 26:585–92. doi: 10.1111/j.1440-1746.2010.06487.x 21332554

[10] Le DT Durham JN Smith KN Wang H Bartlett BR Aulakh LK. Mismatch-Repair Deficiency Predicts Response of Solid Tumors to PD-1 Blockade. Science (2017) 357:409–13. doi: 10.1126/science.aan6733 PMC5576142 28596308

[11] Middha S Zhang L Nafa K Jayakumaran G Wong D Kim HR. Reliable Pan-Cancer Microsatellite Instability Assessment by Using Targeted Next-Generation Sequencing Data. JCO Precis Oncol (2017) 2017. doi: 10.1200/PO.17.00084 PMC6130812 30211344

[12] Le DT Uram JN Wang H Bartlett BR Kemberling H Eyring AD. PD-1 Blockade in Tumors With Mismatch-Repair Deficiency. N Engl J Med (2015) 372:2509–20. doi: 10.1056/NEJMoa1500596 PMC4481136 26028255

[13] Marabelle A Le DT Ascierto PA Di Giacomo AM De Jesus-Acosta A Delord J-P. Efficacy of Pembrolizumab in Patients With Noncolorectal High Microsatellite Instability/Mismatch Repair–Deficient Cancer: Results From the Phase II KEYNOTE-158 Study. JCO (2019) 38:1–10. doi: 10.1200/JCO.19.02105 PMC8184060 31682550

[14] Shitara K Van Cutsem E Bang Y-J Fuchs CS Wyrwicz L KW L. Pembrolizumab With or Without Chemotherapy *vs* Chemotherapy in Patients With Advanced G/GEJ Cancer (GC) Including Outcomes According to Microsatellite Instability-High (MSI-H) Status in KEYNOTE-062. Ann Oncol (2019) 30:v878–9. doi: 10.1093/annonc/mdz394.035

[15] Shitara K Van Cutsem E Bang Y-J Fuchs C Wyrwicz L Lee K-W. Efficacy and Safety of Pembrolizumab or Pembrolizumab Plus Chemotherapy *vs* Chemotherapy Alone for Patients With First-Line, Advanced Gastric Cancer: The KEYNOTE-062 Phase 3 Randomized Clinical Trial. JAMA Oncol (2020) 6:1571–80. doi: 10.1001/jamaoncol.2020.3370 PMC7489405 32880601

[16] Pietrantonio F Randon G Di Bartolomeo M Luciani A Chao J Smyth EC. Predictive Role of Microsatellite Instability for of PD-1 Blockade in Patients With Advanced Gastric Cancer: A Meta-Analysis of Randomized Clinical Trials. ESMO Open (2021) 6:100036. doi: 10.1016/j.esmoop.2020.100036 33460964 PMC7815473

[17] Borcoman E Nandikolla A Long G Goel S Le Tourneau C. Patterns of Response and Progression to Immunotherapy. Am Soc Clin Oncol Educ Book (2018), 38:169–78. doi: 10.1200/EDBK_200643 30231380

[18] Saâda-Bouzid E Defaucheux C Karabajakian A Coloma VP Servois V Paoletti X. Hyperprogression During Anti-PD-1/PD-L1 Therapy in Patients With Recurrent and/or Metastatic Head and Neck Squamous Cell Carcinoma. Ann Oncol (2017) 28:1605–11. doi: 10.1093/annonc/mdx178 28419181

[19] Kas B Talbot H Ferrara R Richard C Lamarque J-P Pitre-Champagnat S. Clarification of Definitions of Hyperprogressive Disease During Immunotherapy for Non–Small Cell Lung Cancer. JAMA Oncol (2020) 6:1039–46. doi: 10.1001/jamaoncol.2020.1634 PMC7290708 32525513

[20] Ji Z Peng Z Gong J Zhang X Li J Lu M. Hyperprogression After Immunotherapy in Patients With Malignant Tumors of Digestive System. BMC Cancer (2019) 19:1–9. doi: 10.1186/s12885-019-5921-9 31315610 PMC6637510

[21] Lai Y-H Yang S. Hyperprogressive Disease After Nivolumab in a Patient With Microsatellite Instability-High Ampullary Cancer. J Cancer Res Pract (2019) 6:50–4. doi: 10.4103/JCRP.JCRP_9_18

[22] Frelaut M du Rusquec P de Moura A Le Tourneau C Borcoman E. Pseudoprogression and Hyperprogression as New Forms of Response to Immunotherapy. BioDrugs (2020) 34:463–76. doi: 10.1007/s40259-020-00425-y 32394415

[23] Le Tourneau C Servois V Diéras V Ollivier L Tresca P Paoletti X. Tumour Growth Kinetics Assessment: Added Value to RECIST in Cancer Patients Treated With Molecularly Targeted Agents. Br J Cancer (2012) 106:854–7. doi: 10.1038/bjc.2012.10 PMC3305968 22281665

[24] Colle R Radzik A Cohen R Pellat A Lopez-Tabada D Cachanado M. Pseudoprogression in Patients Treated With Immune Checkpoint Inhibitors for Microsatellite Instability-High/Mismatch Repair-Deficient Metastatic Colorectal Cancer. Eur J Cancer (2021) 144:9–16. doi: 10.1016/j.ejca.2020.11.009 33316636

[25] Popat V Gerber DE. Hyperprogressive Disease: A Distinct Effect of Immunotherapy? J Thorac Dis (2019) 11:S262–5. doi: 10.21037/jtd.2019.01.97 PMC6424758 30997192

[26] Kim JY Lee KH Kang J Borcoman E Saada-Bouzid E Kronbichler A. Hyperprogressive Disease During Anti-PD-1 (PDCD1)/PD-L1 (CD274) Therapy: A Systematic Review and Meta-Analysis. Cancers (2019) 11:1699. doi: 10.3390/cancers11111699 PMC6896059 31683809

[27] Hamakawa T Nishikawa K Tanaka E Nagae A Toshiyama R Miyo M. Palliative Radiotherapy and Sequential Nivolumab Administration for Recurrent Gastric Cancer-A Case Report. Gan To Kagaku Ryoho (2019) 46:2557–9. 32156997

[28] Huang L-T Ma J-T Zhang S-L Li X-H Sun L Jing W. Durable Clinical Response to Pyrotinib After Resistance to Prior Anti-HER2 Therapy for HER2-Positive Advanced Gastric Cancer: A Case Report. Front Oncol (2019) 9:1453. doi: 10.3389/fonc.2019.01453 31956604 PMC6951398

[29] Ogata T Satake H Ogata M Hatachi Y Yasui H. Hyperprogressive Disease in the Irradiation Field After a Single Dose of Nivolumab for Gastric Cancer: A Case Report. Case Rep Oncol (2018) 11:143–50. doi: 10.1159/000487477 PMC5903152 29681813

[30] Takeoka T Okada K Matsuno H Konishi K Ota H Yokoyama S. [Hyperprogressive Disease During Treatment With Nivolumab for Recurrence of Gastric Cancer]. Gan To Kagaku Ryoho (2020) 47:165–7. 32381893

[31] Togasaki K Sukawa Y Kanai T Takaishi H. Clinical Efficacy of Immune Checkpoint Inhibitors in the Treatment of Unresectable Advanced or Recurrent Gastric Cancer: An Evidence-Based Review of Therapies. Onco Targets Ther (2018) 11:8239–50. doi: 10.2147/OTT.S152514 PMC6254591 30538493

[32] Aoki M Shoji H Nagashima K Imazeki H Miyamoto T Hirano H. Hyperprogressive Disease During Nivolumab or Irinotecan Treatment in Patients With Advanced Gastric Cancer. ESMO Open (2019) 4:e000488. doi: 10.1136/esmoopen-2019-000488 31231567 PMC6555603

[33] Hagi T Kurokawa Y Kawabata R Omori T Matsuyama J Fujitani K. Multicentre Biomarker Cohort Study on the Efficacy of Nivolumab Treatment for Gastric Cancer. Br J Cancer (2020) 123:965–72. doi: 10.1038/s41416-020-0975-7 PMC7492241 32616848

[34] Kamada T Togashi Y Tay C Ha D Sasaki A Nakamura Y. PD-1+ Regulatory T Cells Amplified by PD-1 Blockade Promote Hyperprogression of Cancer. PNAS (2019) 116:9999–10008. doi: 10.1073/pnas.1822001116 31028147 PMC6525547

[35] Sasaki A Nakamura Y Mishima S Kawazoe A Kuboki Y Bando H. Predictive Factors for Hyperprogressive Disease During Nivolumab as Anti-PD1 Treatment in Patients With Advanced Gastric Cancer. Gastric Cancer (2019) 22:793–802. doi: 10.1007/s10120-018-00922-8 30627987

[36] Cristescu R Mogg R Ayers M Albright A Murphy E Yearley J. Pan-Tumor Genomic Biomarkers for PD-1 Checkpoint Blockade–Based Immunotherapy. Science (2018) 362. doi: 10.1126/science.aar3593 PMC6718162 30309915

[37] Llosa NJ Cruise M Tam A Wicks EC Hechenbleikner EM Taube JM. The Vigorous Immune Microenvironment of Microsatellite Instable Colon Cancer Is Balanced by Multiple Counter-Inhibitory Checkpoints. Cancer Discov (2015) 5:43–51. doi: 10.1158/2159-8290.CD-14-0863 25358689 PMC4293246

[38] Marabelle A Fakih M Lopez J Shah M Shapira-Frommer R Nakagawa K. Association of Tumour Mutational Burden With Outcomes in Patients With Advanced Solid Tumours Treated With Pembrolizumab: Prospective Biomarker Analysis of the Multicohort, Open-Label, Phase 2 KEYNOTE-158 Study. Lancet Oncol (2020) 21:1353–65. doi: 10.1016/S1470-2045(20)30445-9 32919526

[39] Kim J Kim B Kang SY Heo YJ Park SH Kim ST. Tumor Mutational Burden Determined by Panel Sequencing Predicts Survival After Immunotherapy in Patients With Advanced Gastric Cancer. Front Oncol (2020) 10:314. doi: 10.3389/fonc.2020.00314 32232003 PMC7082319

[40] Mezquita L Auclin E Ferrara R Charrier M Remon J Planchard D. Association of the Lung Immune Prognostic Index With Immune Checkpoint Inhibitor Outcomes in Patients With Advanced Non-Small Cell Lung Cancer. JAMA Oncol (2018) 4:351–7. doi: 10.1001/jamaoncol.2017.4771 PMC5885829 29327044

[41] Meyers DE Stukalin I Vallerand IA Lewinson RT Suo A Dean M. The Lung Immune Prognostic Index Discriminates Survival Outcomes in Patients With Solid Tumors Treated With Immune Checkpoint Inhibitors. Cancers (2019) 11:1713. doi: 10.3390/cancers11111713 PMC6896022 31684111

[42] Hou B Wang P Liu T Chen S Li T Zhang S. Association of the Pretreatment Lung Immune Prognostic Index With Survival Outcomes in Advanced Gastric Cancer Patients Treated With Immune Checkpoint Inhibitors. Clinics Res Hepatol Gastroenterol (2021) 45:101748. doi: 10.1016/j.clinre.2021.101748 34182184

[43] Auclin E Vuagnat P Smolenschi C Taieb J Alfonso JA Nebot L. 2p Lung Immune Prognostic Index (LIPI) Can Identify the Fast-Progressor to Immune Checkpoints Inhibitors (ICI) in Microsatellite Instability (MSI) or Mismatch Repair Deficient (dMMR) Tumours. Ann Oncol (2020) 31:S1418. doi: 10.1016/j.annonc.2020.10.487

[44] Russo GL Moro M Sommariva M Cancila V Boeri M Centonze G. Antibody–Fc/FcR Interaction on Macrophages as a Mechanism for Hyperprogressive Disease in Non–Small Cell Lung Cancer Subsequent to PD-1/PD-L1 Blockade. Clin Cancer Res (2019) 25:989–99. doi: 10.1158/1078-0432.CCR-18-1390 30206165

[45] Yamaguchi K Tsuchihashi K Tsuji K Kito Y Tanoue K Ohmura H. Prominent PD-L1-Positive M2 Macrophage Infiltration in Gastric Cancer With Hyper-Progression After Anti-PD-1 Therapy. Med (Baltimore) (2021) 100:e25773. doi: 10.1097/MD.0000000000025773 PMC8133284 34106609

[46] Kato S Goodman A Walavalkar V Barkauskas DA Sharabi A Kurzrock R. Hyperprogressors After Immunotherapy: Analysis of Genomic Alterations Associated With Accelerated Growth Rate. Clin Cancer Res (2017) 23:4242–50. doi: 10.1158/1078-0432.CCR-16-3133 PMC5647162 28351930

