# Supplementary material for: Hyperprogressive Disease After Combined Anti-PD-L1 and Anti-CTLA-4 Immunotherapy for MSI-H/dMMR Gastric Cancer: A Case Report
Source: Front Oncol. 2021 Sep 24;11:756365. doi: 10.3389/fonc.2021.756365 (PMC8499695; doi:10.3389/fonc.2021.756365)
Supplement: Supplementary file 1 [file Table_1.docx]

Supplementary Material

| **Mutations** | **Fusion transcripts** | **Expression** |
| --- | --- | --- |
| AKT1 (3) | ALK | ALK |
| ALK (22, 23, 24, 25) | AXL | CCND1 |
| AXL (5, 11, 15, 17) | BRAF | EGFR |
| BRAF (11, 15) | CCND1 | ERBB2 |
| CTNNB1 (3) | EGFR | FGFR1 |
| CYSLTR2 (6) | FGFR1 | FGFR2 |
| DDR2 (17) | FGFR2 | FGFR3 |
| EGFR (18, 19, 20, 21) | FGFR3 | MET |
| ERBB2 (20) | MAP2K1 | NTRK1 |
| FGFR1 (2, 8, 9, 10, 17) | MET | NTRK2 |
| FGFR2 (2, 5, 7, 8, 9, 10) | NRG1 | NTRK3 |
| FGFR3 (3, 5, 8, 9, 10) | NTRK1 | RET |
| GNA11 (4,5) | NTRK2 | ROS1 |
| GNAS (8, 9) | NTRK3 |  |
| GNAQ (4,5) | PPARG |  |
| HRAS (2, 3, 4) | RAF1 |  |
| IDH1 (4) | RET |  |
| IDH2 (4) | ROS1 |  |
| KEAP1 (full) |  |  |
| KIT (11, 13, 17) |  |  |
| KRAS (2, 3, 4) |  |  |
| MAP2K1 (2, 3) |  |  |
| MET (13-19) |  |  |
| NRAS (2, 3, 4) |  |  |
| PIK3CA (9, 20) |  |  |
| POLE (9-14) |  |  |
| RAF1 (4-7, 9-12) |  |  |
| RET (11, 13, 14, 15, 16) |  |  |
| ROS1 (38) |  |  |
| STK11 (full) |  |  |
| TP53 (full) |  |  |

**Supplementary Table 1.** List of genes covered in the RNAseq analysis (exons are detailed only for mutations analysis)
